# Protective Effects of N-Acetylcysteine in Alleviating Cocaine-Mediated Microglial Activation and Neuroinflammation

**DOI:** 10.3390/biology14070893

**Published:** 2025-07-20

**Authors:** Uma Maheswari Deshetty, Abiola Oladapo, Yazhini Mohankumar, Elias Horanieh, Shilpa Buch, Palsamy Periyasamy

**Affiliations:** Department of Pharmacology and Experimental Neuroscience, University of Nebraska Medical Center, Omaha, NE 68198, USA; udeshetty@unmc.edu (U.M.D.); aa.oladapo00@gmail.com (A.O.); yazhinimohan225@gmail.com (Y.M.); ehoranieh@unmc.edu (E.H.); sbuch@unmc.edu (S.B.)

**Keywords:** cocaine, neuroinflammation, microglial activation, N-acetylcysteine, mitophagy, autophagy, lysosomes

## Abstract

Cocaine misuse induces microglial activation and neuroinflammation, contributing to neurodegeneration and behavioral impairments. Prior studies have shown that cocaine induces mitochondrial dysfunction, dysregulated mitophagy, and lysosomal impairment in microglia. Here, we investigated the therapeutic potential of N-acetylcysteine (NAC) in mitigating cocaine-induced microglial activation and neuroinflammation. Mouse primary microglial cells (MPMs) were pretreated with NAC (5 mM) for 1 h prior to cocaine exposure (10 µM, 24 h) and analyzed for markers of microglial activation, mitophagy, and lysosomal integrity using Western blot, Seahorse assays, lysosomal pH, and membrane potential measurements. In vivo, C57BL/6N mice received NAC (200 mg/kg, i.p.) 1 h before daily cocaine injections (20 mg/kg, i.p.) for 7 days. Behavioral assays (open field, novel object recognition) and brain biomarker analyses (frontal cortex, hippocampus) were performed. Cocaine exposure elevated CD11b, mitophagy markers (PINK1, PARK, and DLP1), and autophagy proteins (Beclin1, and p62), while impairing mitochondrial and lysosomal functions. NAC pretreatment restored mitochondrial and lysosomal function, reduced reactive oxygen species, and normalized protein expression. In vivo, NAC also alleviated cocaine-induced microglial activation and behavioral deficits. These findings highlight NAC as a promising therapeutic agent to counteract cocaine-mediated neuroinflammation and neurotoxicity.

## Simple Summary

Cocaine misuse can harm the brain by overstimulating special immune-like cells called microglia. These cells normally protect the brain, but when they become overactive, they release harmful substances that damage brain tissue. This can lead to memory loss, mood changes, and long-term brain problems. In this study, we tested whether a common antioxidant called N-acetylcysteine, which is used in hospitals to treat other medical conditions, could help protect the brain from the harmful effects of cocaine. We used both brain cells cultured in the laboratory and live mice to see how well this treatment worked. We found that N-acetylcysteine reduced brain inflammation, helped restore energy production in brain cells, and restored the cellular autophagy system. Mice treated with N-acetylcysteine also showed better behavior after being exposed to cocaine. These results suggest that this widely available treatment may help reduce brain damage caused by cocaine misuse. This research may lead to new strategies for helping people recover from the effects of cocaine and could also be useful for treating other brain disorders that involve inflammation.

## 1. Introduction

Cocaine, a potent and addictive stimulant derived from the leaves of the *Erythroxylon coca* plant, poses a significant global health burden. In the United States alone, an estimated 5 million individuals reported regular cocaine use within the past year [1], underscoring the persistent prevalence of cocaine use disorder (CUD). Beyond its addictive potential, cocaine use is associated with widespread morbidity, increased healthcare utilization, and a growing number of related fatalities [2]. Although much attention has been directed toward the opioid epidemic, cocaine-related overdose deaths in the United States increased from 1.8 to 8.6 per 100,000 population between 2011 and 2023 [3,4], emphasizing the urgent need for effective therapeutic strategies to address CUD.

The neurobiological effects of cocaine are multifaceted, with its primary action involving the inhibition of dopamine reuptake through blockage of the dopamine transporter [5]. This inhibition leads to excessive dopamine accumulation in synaptic clefts, reinforcing its addictive properties [6]. Cocaine also disrupts the balance of oxidative and antioxidative processes by inhibiting monoamine oxidase, resulting in the overproduction of reactive oxygen species (ROS) and subsequent oxidative stress [6,7]. In the central nervous system (CNS), ROS are pivotal in initiating inflammatory responses, particularly through the activation of microglia, the resident innate immune cells of the CNS [8]. While microglia are essential for maintaining CNS homeostasis, their hyperactivation under pathological conditions, such as during CUD, drives neuroinflammatory cascades that exacerbates neuronal damage and dysfunction [9].

Microglial activation is characterized by the release of pro-inflammatory cytokines, including tumor necrosis factor-alpha (TNF-α), interleukin-1 beta (IL-1β), and interleukin-6 (IL-6). These mediators contribute to a toxic microenvironment by disrupting astrocytic glutamate reuptake, which exacerbates excitotoxicity and neuronal injury [10,11]. Cocaine exposure has been linked to increased production of these pro-inflammatory mediators, activation of astrocytes and microglia, and subsequent neuronal damage [7,12]. Additionally, mitochondrial dysfunction plays a central role in cocaine-induced neurotoxicity [13,14,15]. Cocaine disrupts mitochondrial energy metabolism and impairs mitophagy, the selective autophagic process responsible for the removal of damaged mitochondria [16]. This dysregulation leads to ROS accumulation and primes the NLR family pyrin domain containing 3 (NLRP3) inflammasome, a key driver of microglial activation and neuroinflammation [17].

Another critical aspect of cocaine-induced cellular damage is lysosomal dysfunction. Lysosomes, essential for the degradation of damaged organelles and macromolecules, lose their functional integrity following cocaine exposure. Studies have reported decreased expression of lysosomal-associated membrane protein 2 (LAMP2) and cathepsin D in microglia treated with cocaine, indicative of impaired lysosomal activity [18]. This dysfunction hinders cellular clearance mechanisms and promotes the accumulation of inflammatory mediators, perpetuating a cycle of neuroinflammation and neuronal damage.

Given the central roles of oxidative stress, mitochondrial dysfunction, and lysosomal impairment in cocaine-induced neuroinflammation, therapeutic strategies targeting these pathways are being explored. N-acetylcysteine (NAC), a derivative of the amino acid cysteine, has shown promise as a therapeutic agent due to its potent antioxidant and anti-inflammatory properties [19,20,21,22]. NAC acts as a precursor to glutathione, a critical endogenous antioxidant that neutralizes ROS and restores redox balance [23]. Additionally, NAC inhibits the activation of nuclear factor kappa B (NF-κB), a transcription factor that drives the expression of pro-inflammatory cytokines [24]. These dual actions highlight NAC as a compelling candidate for addressing cocaine-induced microglial activation and neuroinflammation.

Beyond its antioxidant properties, NAC has demonstrated the ability to modulate autophagic and mitophagic pathways, further contributing to its neuroprotective effects [16,18]. NAC has been shown to restore mitochondrial membrane potential, reduce mitochondrial ROS, and enhance mitophagy in neurotoxic models [16]. Furthermore, NAC stabilizes lysosomal pH and improves lysosomal membrane potential, restoring the functional capacity of lysosomes and facilitating the clearance of damaged cellular components [18]. These findings highlight the potential of NAC to address both the upstream and downstream effects of cocaine-induced oxidative stress and neuroinflammation.

Preclinical studies using animal models of cocaine exposure have provided further evidence of the neuroprotective potential of NAC. In these models, cocaine administration induces microglial activation, evidenced by increased expression of activation markers such as CD11b and elevated levels of pro-inflammatory cytokines [17]. Behavioral studies reveal hyperlocomotion and heightened anxiety-like behaviors, paralleling symptoms observed in humans with CUD [17,25]. NAC treatment in these models reduces microglial activation, suppresses inflammatory mediator expression, and normalizes behavioral alterations, underscoring its translational potential for treating cocaine-induced neurotoxicity.

Despite promising preclinical findings, clinical trials investigating the efficacy of NAC in treating CUD have encountered challenges, including variability in treatment responses and adherence [26]. This highlights the need for continued research to optimize the therapeutic applications of NAC and clarify its mechanisms of action. By addressing oxidative stress, mitochondrial dysfunction, and lysosomal impairment, NAC offers a multifaceted approach to mitigating the neuroinflammatory consequences of cocaine use.

The present study aims to evaluate the therapeutic potential of NAC in alleviating cocaine-induced neuroinflammation and neurotoxicity. By elucidating the protective mechanisms of NAC, this study seeks to fill critical gaps in our understanding of cocaine-induced neurotoxicity and inform the development of targeted therapeutic strategies for CUD. These findings could also have broader implications for addressing other neuroinflammatory conditions associated with substance abuse.

## 2. Materials and Methods

### 2.1. Chemicals and Reagents

Antibodies targeting CD11b (Novus Biologics, Centennial, CO, USA, Cat. No. NB110-89474), Beclin-1 (Santa Cruz Biotechnology, Inc., Dallas, TX, USA, Cat. No. sc-11427), p62 (MBL International, Schaumburg, IL, USA, Cat. No. PM045), LC3B-II (Novus Biologicals, Centennial, CO, USA, Cat. No. NB100-2220), PINK1 (Cell Signaling Technology, Inc., MA, USA, Cat. No. 6946S), Parkin (Santa Cruz Biotechnology, Inc., Dallas, TX, USA, Cat. No. sc-32282), DLP1 (BD Biosciences, Franklin Lakes, NJ, USA, Cat. No. 611112), Optineurin (Santa Cruz Biotechnology, Inc., Dallas, TX, USA, Cat. No. sc-271549), Peroxidase-AffiniPure Goat Anti-Rabbit IgG (H + L) (Jackson ImmunoResearch Inc., West Grove, PA, USA, Cat. No. 111035-003), and Peroxidase-AffiniPure Goat Anti-Mouse IgG (H + L) (Jackson ImmunoResearch Inc., Cat. No. 115-035-003) were procured from commercial vendors as specified. Reagents used in cell culture included Dulbecco’s Modified Eagle Medium (DMEM, Corning, Oneonta, NY, USA, Cat. No. 10-013-CV), heat-inactivated fetal bovine serum (FBS, Atlanta Biologicals, Flowery Branch, GA, USA, Cat. No. S11150H), and penicillin-streptomycin (Life Technologies, Carlsbad, CA, USA, Cat. No. 15140-122). All other chemicals were of analytical grade procured from Thermo Fischer Scientific, Waltham, MA, USA.

### 2.2. Cell Culture and Treatments

Mouse primary microglia (MPMs) were isolated from the cortices of postnatal day 1–3 C57BL/6N mice. Briefly, the cortices were dissected, minced, and enzymatically digested with 0.25% trypsin-EDTA at 37 °C for 10 min. The dissociated cells were passed through a 40 μM cell strainer and seeded onto T75 flasks in DMEM supplemented with 10% fetal bovine serum (FBS) and 1% penicillin-streptomycin. After 7–10 days, MPMs were harvested by mild shaking and cultured in 6-well plates at a density of 0.5 × 10^6^ cells per well using DMEM supplemented with 10% heat-inactivated FBS, penicillin-streptomycin (Invitrogen, Carlsbad, CA, USA, 15070063), granulocyte-macrophage colony-stimulating factor (Sigma-Aldrich, St. Louis, MO, USA, G0282), and OPI media supplement (Sigma-Aldrich, O5003-5 mL). The cells were serum-starved for 8 h before being pretreated with NAC (5 mM) for 1 h, followed by exposure to cocaine (10 µM) for 24 h.

### 2.3. Animal Studies

Male C57BL/6N mice were procured from Charles River Laboratories (Wilmington, MA, USA). The animals were housed under standardized conditions with controlled temperature and humidity, maintained on a 12 h light/dark cycle, and provided with food and water ad libitum. All experimental procedures adhered to protocols approved by the Institutional Animal Care and Use Committee at the University of Nebraska Medical Center. The mice were randomly divided into four groups (n = 6 per group): 1. saline control, 2. cocaine only (20 mg/kg/day, i.p.), 3. NAC pretreated (200 mg/kg/day, i.p.), and 4. NAC + cocaine. The treatments were administered once daily for 7 consecutive days. Behavioral testing was conducted 1 h after the final injection, after which the mice were sacrificed, and the frontal cortex and hippocampus were dissected for biochemical and molecular analyses.

### 2.4. Western Blotting

Protein lysates from the control and treated MPMs and brain tissues were prepared using RIPA buffer containing phosphatase (Thermo Fisher Scientific, Pittsburgh, PA, USA, Cat. No. 78426) and protease inhibitor cocktails (Thermo Fisher Scientific, Cat. No. 78429). Protein concentrations were determined via a BCA assay (Thermo Fisher Scientific, Pittsburgh, PA, USA, Cat. No. 23227). Equal amounts of protein were separated on sodium dodecyl sulfate-polyacrylamide gel electrophoresis (SDS-PAGE) gels and transferred to polyvinylidene fluoride membranes. Membranes were blocked with 5% non-fat dry milk in Tris-buffered saline with 0.1% Tween-20 (TBST) and incubated overnight at 4 °C with primary antibodies against CD11b, Beclin-1, LC3B-II, p62, PINK1, Parkin, DLP1, and Optineurin. Blots were probed with appropriate HRP-conjugated secondary antibodies, visualized using enhanced chemiluminescence, and quantified using ImageJ software version 5.4p [27]. β-actin was used as internal control in all these experiments.

### 2.5. Measurement of Mitochondrial Membrane Potential

Mitochondrial membrane potential changes in MPMs exposed to NAC, and cocaine were evaluated using the JC-1 Mitochondrial Membrane Potential Assay Kit (Cayman Chemicals, Ann Arbor, MI, USA, 10009172), following the manufacturer’s protocol. In brief, MPMs were seeded at a density of 0.05 × 10^6^ cells per well in a 96-well plate. After exposure to drugs, the cells were treated with JC-1 reagent (100 µL/mL of medium), diluted in serum-free culture medium (1:10 dilution), and incubated for 20 min at 37 °C in a 5% CO_2_ incubator. Subsequently, the cells were washed once with the 1× assay buffer provided in the kit. Fluorescence intensities of JC-1 aggregates (λexcitation = 535 nm; λemission = 585 nm) and monomers (λexcitation = 485 nm; λemission = 535 nm) were measured using a Synergy™ Mx Monochromator-Based Multi-Mode Microplate Reader (BioTek Instruments, Inc., Winooski, VT, USA). Additionally, MPMs were also seeded in 24 well plates (0.2 × 10^6^ cells per well), the cells were treated with drugs and stained with JC1 dye per manufacturer’s instructions. After incubation of 20 min, cells were washed with HBSS buffer (twice). Imaging was performed using Zeiss Observer Z1 inverted microscope (Carl Zeiss, Thornwood, NY, USA).

### 2.6. Measurement of Mitochondrial Superoxide Radicals

The production of mitochondrial superoxide radicals was assessed using the MitoSOX™ Red dye (Thermo Fisher Scientific, Pittsburgh, PA, USA, Cat. No. M36008), which specifically targets mitochondria and fluoresces upon oxidation by superoxide. MPMs were seeded on coverslips placed in 24-well plates at a density of 0.2 × 10^6^ cells per well and treated as described earlier in Section 2.2. Following 24 h of incubation, cells were washed with PBS and stained with 5 μM MitoSOX™ Red dye for 15 min at 37 °C. After staining, the cells were washed again and imaged using a Zeiss Observer Z1 inverted microscope (Carl Zeiss, Thornwood, NY, USA). Imaging was performed within 10–20 min of staining to minimize nuclear accumulation of the dye, which typically begins around 40 min post-staining.

### 2.7. Seahorse XF96 Mitochondrial Stress Test

Mitochondrial oxygen consumption rate (OCR) and extracellular acidification rate (ECAR) were measured using a Seahorse XFp or XFe96 Extracellular Flux Analyzer (Seahorse Bioscience, Billerica, MA, USA). MPMs were seeded in Seahorse culture plates at a density of 0.05 × 10^6^ cells per well in DMEM supplemented with 10% heat-inactivated FBS. After pretreatment with NAC (5 mM) and cocaine (10 µM) for 24 h, the culture medium was replaced with unbuffered DMEM containing 10 mM glucose, 2 mM pyruvate, and 2 mM L-glutamine. Seahorse Flux cartridges were hydrated overnight in a non-CO_2_ incubator using an XF Calibrant solution. OCR was assessed through sequential injections of mitochondrial inhibitors: oligomycin (10 μM), FCCP (20 μM), and rotenone/antimycin A (10 μM). The data were normalized to total protein content (determined by BCA method) and analyzed using Seahorse Wave 2.2.0 software (Seahorse Bioscience).

### 2.8. Lysosomal Membrane Permeability Assay

Lysosomal membrane permeability (LMP) was assessed using acridine orange, a versatile fluorescent dye that readily penetrates cell membranes and accumulates reversibly in acidified, membrane-bound compartments such as lysosomes. MPMs were plated in 96-well plates at a density of 0.05 × 10^6^ cells per well and treated as described earlier in Section 2.2. After incubation, the media were discarded, and acridine orange was added at a concentration of 5 μg/mL, followed by a 15 min incubation at 37 °C. Fluorescence intensity was measured using a Synergy™ Mx Monochromator-Based Multi-Mode Microplate Reader (BioTek Instruments, Inc., Winooski, VT, USA), with excitation at 485 nm and emissions recorded at 530 nm and 620 nm. The data were analyzed to assess LMP and represented as relative fold changes compared to control cells.

### 2.9. Lysosomal pH Detection

MPMs were seeded at a density of 0.2 × 10^6^ cells per well on coverslips in a 24-well plate and transfected overnight with a pFUGW-FIRE-pHLy plasmid (Addgene #170774, Watertown, MA, USA) using OptiMEM medium and Lipofectamine 3000 reagent, as per the manufacturer’s instructions, to measure lysosomal pH levels. After transfection, the medium was replaced with a fresh DMEM starvation medium without FBS, and the cells were treated with NAC and cocaine, as described in Section 2.2, for 24 h. Following treatment, the cells were washed with PBS, and the coverslips were mounted on glass slides with DAPI. Fluorescence images were captured using a fluorescence microscope (BioTek Instruments, Inc., Winooski, VT, USA) with excitation wavelengths of 488 nm (mTFP1) and 594 nm (mCherry). The mCherry fluorescence intensity, representing the pH-insensitive cytosolic domain of the reporter, remained constant across all groups. In contrast, the mTFP1 fluorescence intensity, which is pH-sensitive, varied, reflecting changes in lysosomal pH. The mTFP1/mCherry ratio was calculated and compared to a standard curve to determine lysosomal pH values, as described in a previous publication [28].

### 2.10. Behavioral Assessments

The Open Field Test (OFT) and Novel Object Recognition (NOR) test were conducted as separate trials one hour after drug administration to evaluate locomotor activity, exploratory behavior, and anxiety-like behavior in mice. In the OFT, mice were placed in an open box measuring 50 cm × 50 cm × 38 cm for 30 min. During this period, their locomotor paths were tracked using a video tracking system [25]. The total distance traveled was recorded and analyzed using AnyMaze software (Version 7, Stoelting Co., Wood Dale, IL, USA). For the NOR test, mice were placed in the same OFT chamber, initially with two identical objects for three trials of 10 min each, conducted at intervals of 1.5 h. After the third trial, one of the known objects was replaced with a novel object, and the mice were subjected to an additional 10 min trial. To minimize potential side bias, the positions of the known and novel objects were counterbalanced across animals during the OFT. Locomotor paths were tracked using the video tracking system to evaluate the object recognition behavior of mice. As in the OFT, the locomotor data were recorded and analyzed using AnyMaze software (Version 7, Stoelting Co., Wood Dale, IL, USA) to calculate the discrimination index.

### 2.11. Statistical Analysis

Data is presented as mean ± SEM. Statistical significance was determined based on the experimental design using GraphPad Prism software (version 10.3.1). For comparisons among multiple groups, a nonparametric Kruskal–Wallis one-way ANOVA followed by Dunn’s post hoc test was employed. Pairwise comparisons between two groups were analyzed using the Wilcoxon matched-pairs signed-rank test. For in vivo comparisons between groups, ordinary one-way ANOVA followed by Sidak’s multiple comparison test was utilized. A *p* < 0.05 was considered statistically significant.

## 3. Results

### 3.1. NAC Attenuates Cocaine-Mediated Mito/Autophagy Dysregulation in MPMs

**Figure 1:**
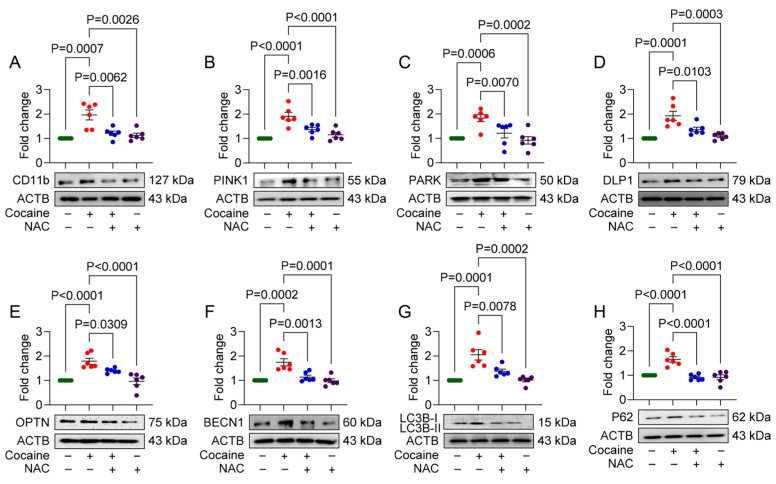
NAC attenuates cocaine-induced cellular activation, mitophagy, and autophagy in MPMs. Representative Western blot showing expressions of the (**A**) microglial activation marker (CD11b); mitophagy markers, namely (**B**) PINK1, (**C**) PARK, (**D**) DLP1, and (**E**) OPTN; and autophagy markers, namely (**F**) BECN1, (**G**) LC3B, and (**H**) P62 in MPMs pre-treated with NAC (5 mM) for 1 h, followed by treatment with cocaine (10 µM) for 24 h. The data are presented as mean ± SEM from six independent experiments. The value of *p* < 0.05 was considered statistically significant. The original western blot images can be found in Supplementary Figure S1.

To investigate the effects of NAC on cocaine-induced microglial activation and its impact on mito/autophagy, we pretreated MPMs with NAC (5 mM) for 1 h, followed by exposure to cocaine (10 µM) for 24 h. Western blot analysis was performed to assess the expression of microglial activation marker (CD11b), mitophagy markers (PINK1, Parkin, DLP1, and optineurin), and autophagy markers (BECN1, LC3B, and P62). Cocaine exposure led to a significant increase in the expression of CD11b, PINK1, Parkin, DLP1, and optineurin, indicating microglial activation and mitophagy dysregulation (Figure 1A–E). NAC pretreatment effectively restored these protein levels to baseline values. Similarly, cocaine exposure elevated the expression of autophagy markers BECN1, LC3B, and P62, while NAC pretreatment normalized these levels (Figure 1F–H). These findings suggest that NAC mitigates cocaine-induced mito/autophagy dysregulation in microglial cells.

### 3.2. NAC Alleviates Cocaine-Mediated Mitochondrial Dysfunction

**Figure 2:**
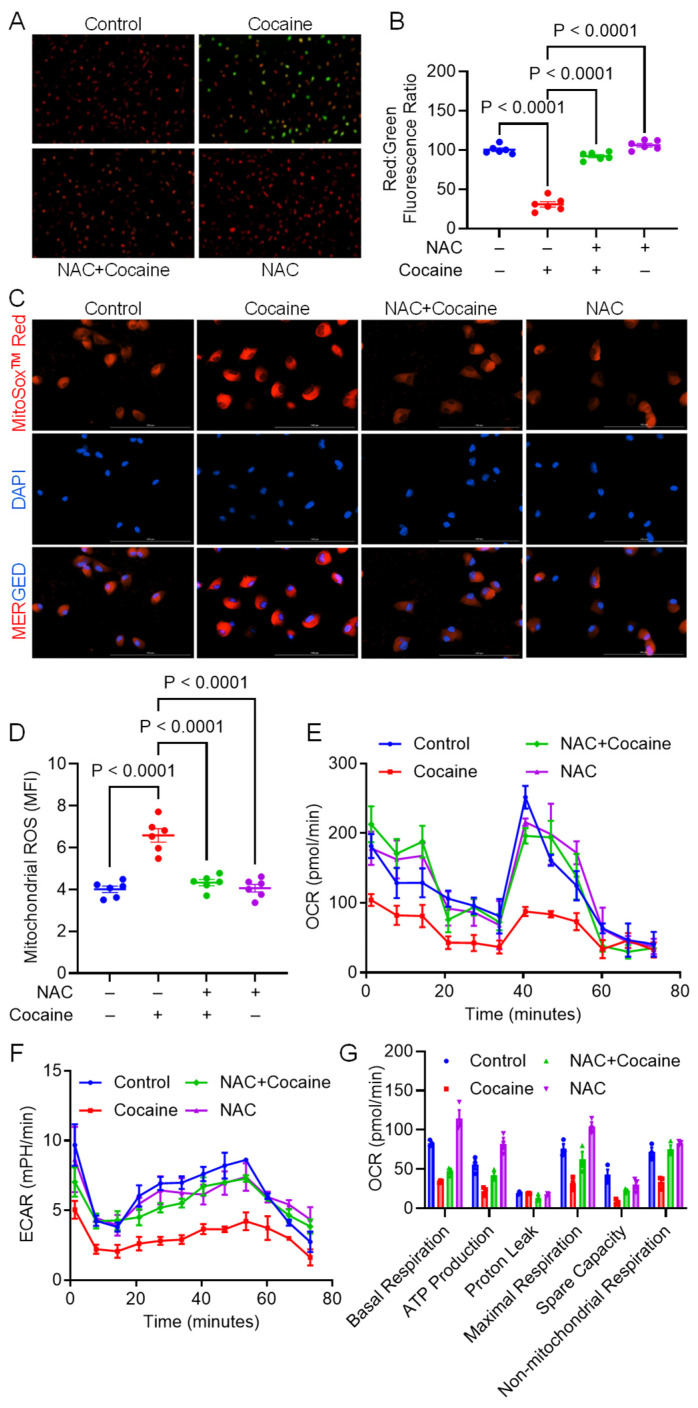
NAC alleviates cocaine-induced mitochondrial dysfunction in MPMs. (**A**) Representative florescence microscopy images and (**B**) quantification of mitochondrial membrane potential; (**C**) representative florescence microscopy images (scale bar: 100 μm) and (**D**) quantification of mitochondrial ROS in MPMs pre-treated with NAC (5 mM) for 1 h, followed by treatment with cocaine (10 µM) for 24 h. Graphs depicting mitochondrial functional analysis by Seahorse XFp Extracellular Flux analyzer with mitochondrial parameters (**E**) OCR, (**F**) ECAR, and (**G**) other mitochondrial parameters calculated based on (**E**) in MPMs pre-treated with NAC (5 mM) for 1 h, followed by treatment with cocaine (10 µM) for 24 h. The data are presented as mean ± SEM from six independent experiments. The value of *p* < 0.05 was considered statistically significant.

Building on the observation that NAC counteracted cocaine-induced mito/autophagy dysregulation, we investigated its effect on mitochondrial function. Mitochondrial membrane potential (Δψm) was assessed using the JC-1 assay. In intact mitochondria, JC-1 accumulates and forms aggregates, resulting in the emission of bright red fluorescence. In contrast, in depolarized mitochondria, the dye remains as monomers, which emit green fluorescence. Cocaine exposure led to a significant decrease in Δψm in MPMs, as indicated by a reduced red-to-green fluorescence ratio. However, NAC treatment restored the Δψm (Figure 2A,B). Mitochondrial ROS levels were measured using MitoSOX staining, and mitochondrial function was assessed using Seahorse analysis. Cocaine exposure significantly increased mitochondrial ROS levels, as evidenced by an elevated mean fluorescence intensity (MFI). However, NAC pretreatment significantly reduced MFI, indicating its protective role against mitochondrial oxidative stress (Figure 2C,D). Mitochondrial function was further assessed by measuring the ECAR and OCR using the Seahorse XFp Extracellular Flux Analyzer. Cocaine-exposed MPMs exhibited significant reductions in OCR, ECAR, and key mitochondrial parameters, including basal respiration, ATP production, maximal respiration, and spare respiratory capacity (Figure 2E–G). In contrast, NAC pretreatment restored OCR, ECAR, and other mitochondrial respiratory parameters to near-control levels. These findings demonstrate that NAC effectively mitigates cocaine-induced mitochondrial dysfunction.

### 3.3. NAC Ameliorates Cocaine-Mediated Lysosomal Dysfunction

**Figure 3:**
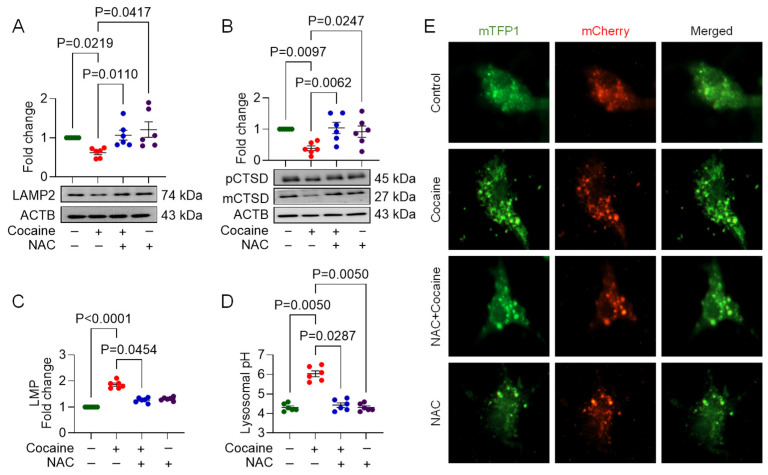
NAC ameliorates cocaine-induced lysosomal dysfunction in MPMs. Representative Western blot showing expression of lysosomal markers, namely (**A**) LAMP2, and (**B**) cathepsin D (CTSD), precursor CTSD (pCTSD), mature CTSD (mCTSD); graph showing (**C**) lysosomal membrane potential (LMP), (**D**) quantification and (**E**) representative florescence images demonstrating changes in lysosomal pH in MPMs pre-treated with NAC (5 mM) for 1 h, followed by treatment with cocaine (10 µM) for 24 h. The data are presented as mean ± SEM from six independent experiments. The value of *p* < 0.05 was considered statistically significant. The original western blot images can be found in Supplementary Figure S2.

Given the critical role of lysosomes in cellular homeostasis, we next assessed the ability of NAC to alleviate cocaine-induced lysosomal dysfunction. Lysosomal-specific protein markers LAMP2 and cathepsin D were evaluated using Western blot analysis, while LMP and lysosomal pH were measured using acridine orange staining and the FIRE-pHLy biosensor, respectively. Cocaine exposure reduced the expression of LAMP2 and cathepsin D, indicative of lysosomal dysfunction (Figure 3A,B). NAC pretreatment restored these protein levels to control values. Cocaine exposure also increased LMP and lysosomal pH, further confirming lysosomal dysfunction (Figure 3C–E). NAC pretreatment reversed these changes, normalizing both LMP and lysosomal pH, suggesting that NAC protects against cocaine-induced lysosomal damage.

### 3.4. Protective Effect of NAC on Cocaine-Induced Molecular and Behavioral Alterations in Mice

**Figure 4:**
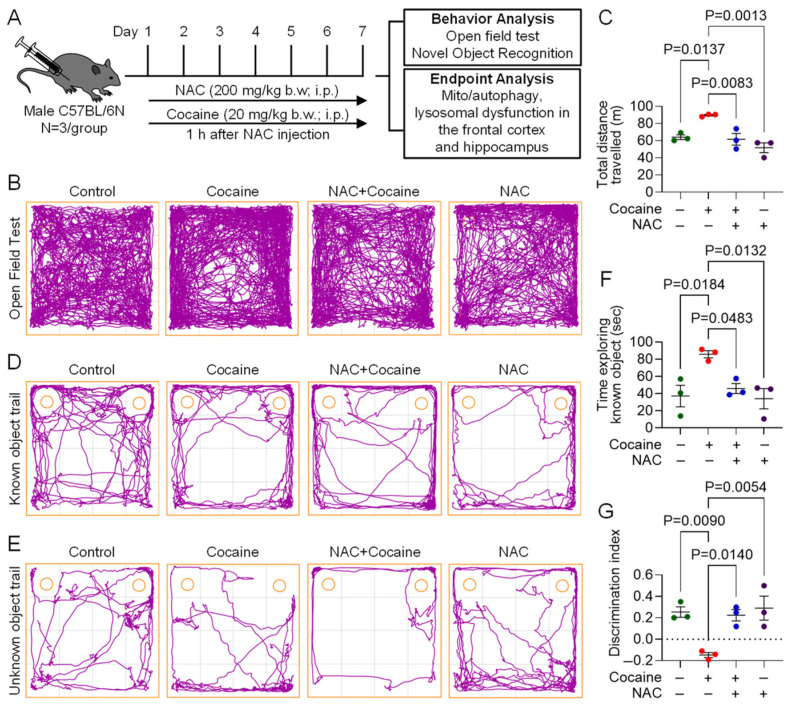
Protective effects of NAC on cocaine-induced behavioral changes in mice. (**A**) Line diagram representing the experimental plan. (**B**) Representative track plots showing the movement of mice and (**C**) quantification of total distance traveled by mice in open field apparatus. (**D**) Representative track plots exploratory behavior of mice towards both known objects, and with the known object (right side) replaced by novel object (**E**), and quantification of (**F**) time spent by mice with known object, and (**G**) discrimination index in mice on the 7th day post administration with NAC (200 mg/kg) for 1 h, followed by cocaine (20 mg/kg). The data are presented as mean ± SEM from three mice. The value of *p* < 0.05 was considered statistically significant.

**Figure 5:**
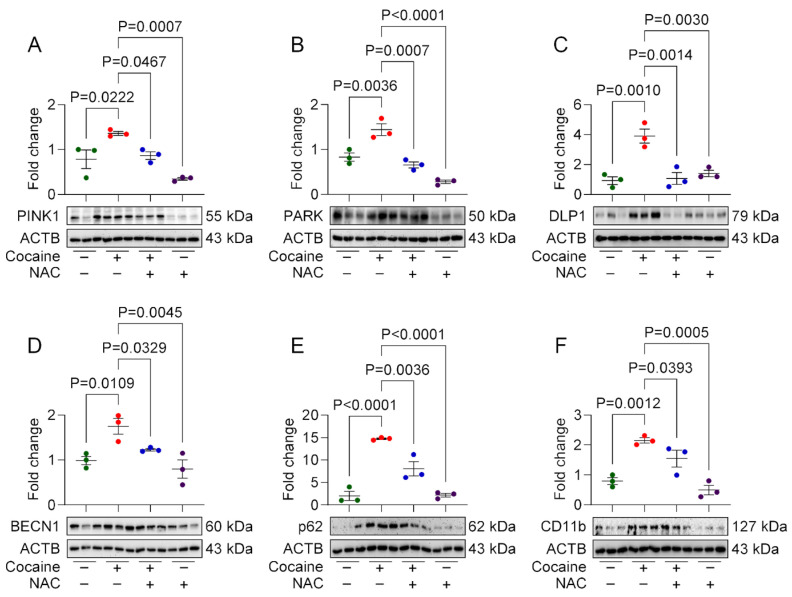
Protective effects of NAC on cocaine-induced molecular changes in mice frontal cortex. Representative Western blot showing expression of mitophagy markers, namely (**A**) PINK1, (**B**) PARK, (**C**) DLP1; autophagy markers, namely (**D**) BECN1, (**E**) P62; and microglial activation marker (**F**) CD11b in frontal cortex region of the male mice brain administered with NAC (200 mg/kg) for 1 h, followed by cocaine (20 mg/kg) for 7 consecutive days. The data are presented as mean ± SD from three mice. The value of *p* < 0.05 was considered statistically significant. The original western blot images can be found in Supplementary Figure S3.

**Figure 6:**
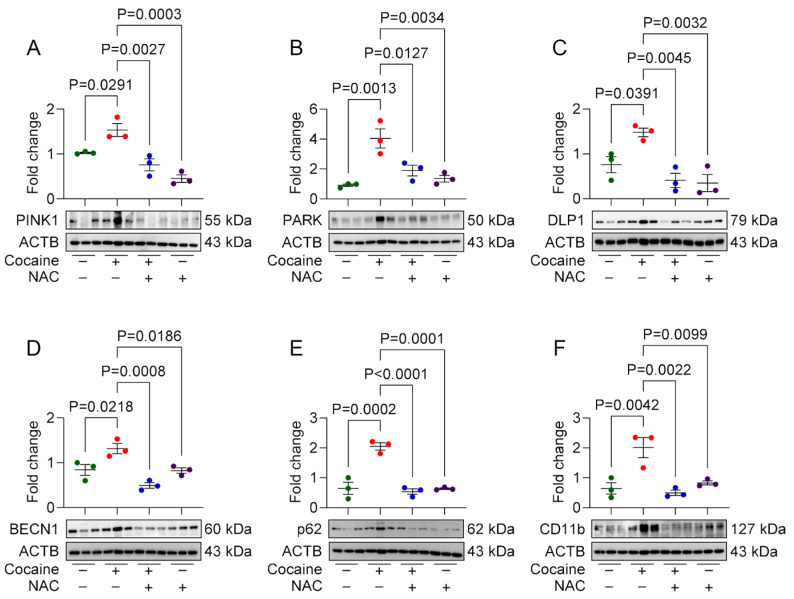
Protective effects of NAC on cocaine-induced molecular changes in mice hippocampus. Representative Western blot showing expression of mitophagy markers, namely (**A**) PINK1, (**B**) PARK, and (**C**) DLP1; autophagy markers, namely (**D**) BECN1, (**E**) P62; and microglial activation marker, (**F**) CD11b in hippocampus region of the male mice brain administered with NAC (200 mg/kg) for 1 h, followed by cocaine (20 mg/kg) for 7 consecutive days. The data are presented as mean ± SD from three mice. The value of *p* < 0.05 was considered statistically significant. The original western blot images can be found in Supplementary Figure S4.

To validate the in vitro findings, we investigated the protective effects of NAC in a mouse model of cocaine exposure. Male C57BL/6N mice were pretreated with NAC (200 mg/kg, i.p.) 1 h prior to daily cocaine injections (20 mg/kg, i.p.) for seven consecutive days. Behavioral assessments, including the OFT and NOR test, were performed on day 7, followed by molecular analysis of brain tissues. In behavioral assays, cocaine-administered mice displayed locomotor hyperactivity, evidenced by increased total distance traveled in the OFT. Additionally, these mice spent significantly less time in the center of the arena, indicating anxiety-like behavior. NAC-pretreated mice exhibited locomotor activity and exploratory behavior comparable to saline-treated controls (Figure 4A–C), demonstrating its ameliorative effect on cocaine-induced behavioral changes. In the NOR test, cocaine-administered mice exhibited impaired object recognition, spending more time exploring familiar objects and showing a negative discrimination index (DI), indicative of cognitive deficits. NAC-pretreated mice, however, spent comparable amounts of time exploring both familiar and novel objects, with a positive DI similar to saline-treated controls (Figure 4D–G). Western blot analysis of the frontal cortex and hippocampus also showed increased expression of CD11b, PINK1, Parkin, DLP1, BECN1, LC3B, and P62 in cocaine-administered mice, indicative of microglial activation, and mito/autophagy dysregulation. These brain regions were chosen based on their critical role in memory, emotion, and reward pathways, which are integral to the structural alterations and cognitive impairments induced by cocaine [29,30]. NAC pretreatment restored the expression of these markers to near-control levels in both frontal cortex (Figure 5A–F), and hippocampus (Figure 6A–F) brain regions.

## 4. Discussion

This study demonstrates the protective effects of NAC against cocaine-induced microglial activation and neuroinflammation by restoring mitochondrial and lysosomal function. Our findings reveal that cocaine exposure dysregulates mitophagy and autophagy processes in microglia, leading to mitochondrial dysfunction, and impairs lysosomal integrity. Notably, pretreatment with NAC prevented these cocaine-induced effects, both in vitro and in vivo. Moreover, NAC alleviated cocaine-induced behavioral impairments in mice, including locomotor hyperactivity and anxiety-like behaviors. These results underscore the potential of NAC as a therapeutic candidate for mitigating neuroinflammation, and neurotoxicity associated with CUD.

Microglia, the principal immune cells of the CNS, play critical roles in tissue repair, neurogenesis, and modulation of immune responses [31,32,33]. However, persistent activation of microglia, such as that observed in response to cocaine exposure, contributes to neurodegenerative processes by releasing pro-inflammatory cytokines and chemokines [9,34,35,36,37]. Previous studies have shown that cocaine triggers innate immune signaling in microglia via the activation of nuclear factor kappa B (NF-κB) and pattern recognition receptors such as toll-like receptor (TLR) 2 and TLR4 [35,37,38]. This pro-inflammatory response exacerbates oxidative stress and neuronal damage, perpetuating the cycle of neuroinflammation. Our findings extend previous research by demonstrating that cocaine-induced microglial activation is closely linked to dysregulated mitophagy and autophagy processes. Mitophagy is critical for clearing damaged mitochondria, a process mediated by proteins such as PINK1, Parkin, and optineurin, which recruit autophagic machinery proteins like Beclin1, LC3B, and p62 [39,40]. In the current study, cocaine exposure elevated the expression of these mitophagy and autophagy markers, indicating impaired mitochondrial clearance. Dysregulated mitophagy leads to the accumulation of dysfunctional mitochondria, which in turn exacerbates ROS production and amplifies inflammatory responses [41,42]. NAC pretreatment restored the expression of these proteins, suggesting its ability to prevent cocaine-induced mitophagy and autophagy dysregulation.

Mitochondrial dysfunction is a hallmark of cocaine-induced cellular toxicity [14]. Cocaine impairs mitochondrial respiration and metabolism, resulting in excessive ROS production and subsequent inflammatory signaling [14,43,44]. Consistent with previous studies, we observed increased ROS levels and significant reductions in mitochondrial OCR and ECAR in cocaine-exposed microglia. These findings align with prior reports linking cocaine to mitochondrial dysfunction in both neural and non-neural tissues [14,15,45]. Remarkably, NAC pretreatment restored OCR, ECAR, and other mitochondrial respiratory parameters to near-control levels, underscoring its potential to mitigate mitochondrial dysfunction through its antioxidant properties.

The lysosomal pathway, which plays a pivotal role in cellular clearance and homeostasis, was also disrupted by cocaine exposure in this study. Cocaine exposure led to decreased expression of lysosomal markers LAMP2 and cathepsin D, increased LMP, and altered lysosomal pH, indicative of lysosomal dysfunction. These findings corroborate previous studies linking lysosomal impairment to inflammation and neurodegenerative processes [18,46,47]. NAC pretreatment preserved lysosomal integrity by normalizing LAMP2 and cathepsin D levels, stabilizing lysosomal pH, and reducing LMP, highlighting its preventive effects against cocaine-induced lysosomal dysfunction.

Our in vivo experiments further demonstrated the prophylactic effects of NAC on cocaine-induced CNS alterations in a mouse model. Cocaine administration resulted in increased microglial activation, dysregulated mitophagy and autophagy, and behavioral deficits. Elevated expression of microglial activation marker (CD11b), mitophagy markers (PINK1, Parkin, and DLP1), and autophagy markers (Beclin1, LC3B, and p62) in the frontal cortex and hippocampus of cocaine-administered mice confirmed the disruption of these pathways. However, NAC pretreatment preserved these protein levels in both the brain regions. Behavioral analyses revealed that cocaine-induced locomotor hyperactivity, anxiety-like behavior, and impaired object recognition in mice. Whereas mice pretreated with NAC showed behavioral patterns similar to saline administered mice. These findings suggest that the neuroprotective effects of NAC extend beyond molecular restoration to include improvements in behavioral outcomes.

While our study demonstrates the prophylactic effects of NAC against cocaine-induced microglial activation and associated dysfunctions, several limitations should be acknowledged. First, the in vitro experiments utilized isolated MPMs, and while these models provide valuable insights, they may not fully replicate the complex cellular interactions and signaling dynamics present in the human CNS. Second, the in vivo mouse model, despite providing valuable insights, does not entirely capture the multifaceted nature of human CUD, including genetic, environmental, and psychosocial factors. Also, the number of animals used for various analyses was limited, potentially affecting statistical power. Increasing the sample size will be an important consideration in our future studies. Additionally, the dosing regimen and route of NAC administration in animal models may not directly translate into optimal therapeutic strategies in humans. Notably, despite promising preclinical findings, NAC has shown limited efficacy in clinical settings for treating CUD [48]. This discrepancy may be attributed to factors such as inadequate dosing, poor bioavailability, variability in patient populations, and the complex pathophysiology of addiction that extends beyond oxidative stress and neuroinflammation. Further research is necessary to address these translational challenges and optimize the pharmacokinetic properties of NAC to enhance its bioavailability and therapeutic efficacy in humans. Combining NAC with other pharmacological agents targeting different aspects of CUD pathophysiology may offer a more comprehensive treatment approach. Additionally, clinical trials with larger, diverse cohorts are essential to evaluate the effectiveness of NAC across various stages of addiction and recovery. Investigating the genetic and molecular factors influencing individual responses to NAC could further personalize treatment strategies, potentially improving outcomes for those affected by CUD. Future studies should also explore the inclusion of female mice, as the use of only male mice in this study represents a limitation.

## 5. Conclusions

This study highlights the therapeutic potential of NAC in mitigating cocaine-induced microglial activation and neuroinflammation. By preserving mitochondrial and lysosomal function, NAC effectively reduces oxidative stress and neuroinflammatory signaling, offering a multifaceted approach to addressing the neurotoxic effects of cocaine. These findings support further exploration of NAC as a potential treatment for CUD and other neuroinflammatory conditions linked to substance abuse. Future studies should focus on the translational potential of NAC in clinical settings and its integration into treatment strategies for CUD.

## Data Availability

The data is contained within the article or Supplementary Material.

## Supplementary Materials

The following supporting information can be downloaded at: https://www.mdpi.com/article/10.3390/biology14070893/s1, Figure S1: WB original images of Figure 1; Figure S2: WB original images of Figure 3; Figure S3: WB original images of Figure 5; Figure S4: WB original images of Figure 6.

## Abbreviations

The following abbreviations are used in this manuscript:

CNS	Central nervous system
CUD	Cocaine use disorder
ECAR	Extracellular acidification rate
IL	Interleukin
LAMP2	Lysosomal associated membrane protein 2
LMP	Lysosomal membrane permeability
MPM	Mouse primary microglia
NAC	N-acetylcysteine
NLRP3	NLR family pyrin domain containing 3
NOR	Novel object recognition rate
OCR	Oxygen consumption rate
OFT	Open field test
TNF-α	Tumor necrosis factor-alpha
TLR	Toll like receptor
