# Supplementary material for: Protective Effects of N-Acetylcysteine in Alleviating Cocaine-Mediated Microglial Activation and Neuroinflammation
Source: Biology (Basel). 2025 Jul 20;14(7):893. doi: 10.3390/biology14070893 (PMC12292458; doi:10.3390/biology14070893)

Protective effects of N-acetylcysteine in alleviating cocaine-mediated microglial activation and neuroinflammation

Uma Maheswari Deshetty[1], Abiola Oladapo[1], Yazhini Mohankumar[1], Elias Horanieh[1], Shilpa Buch[1], Palsamy Periyasamy[1*]

Supplementary Figure S1

Figure 1A

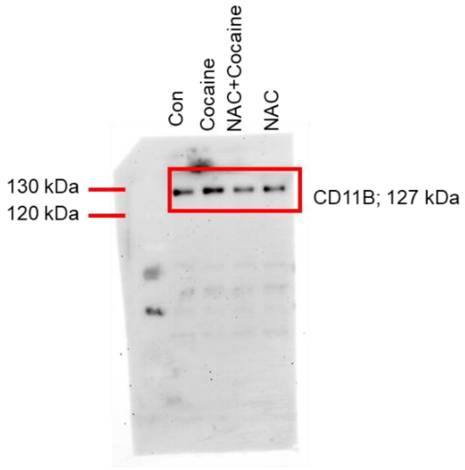

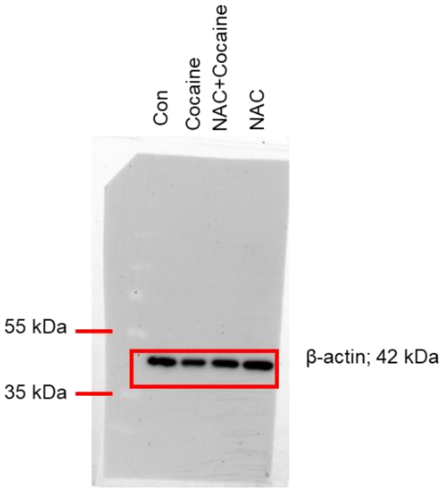

Figure 1B

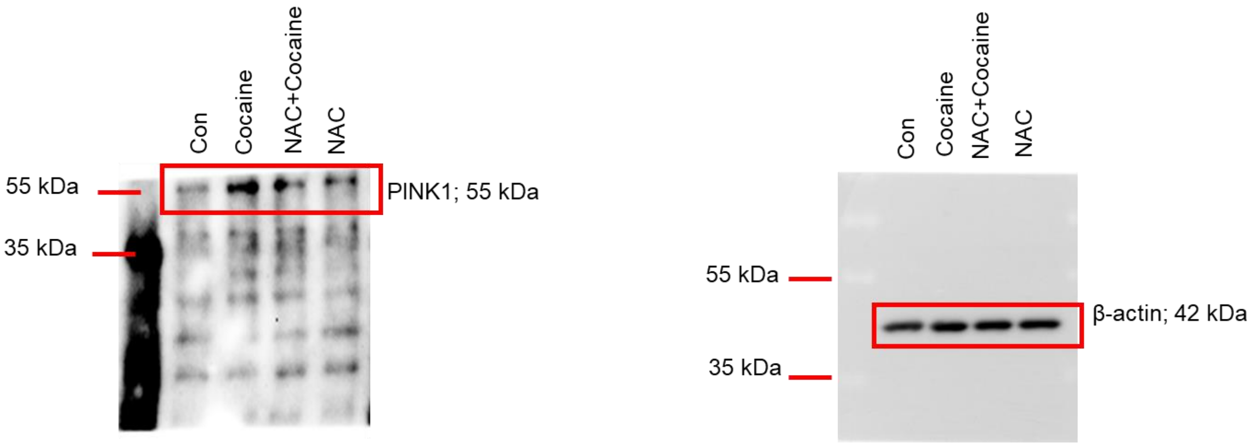

Figure 1C

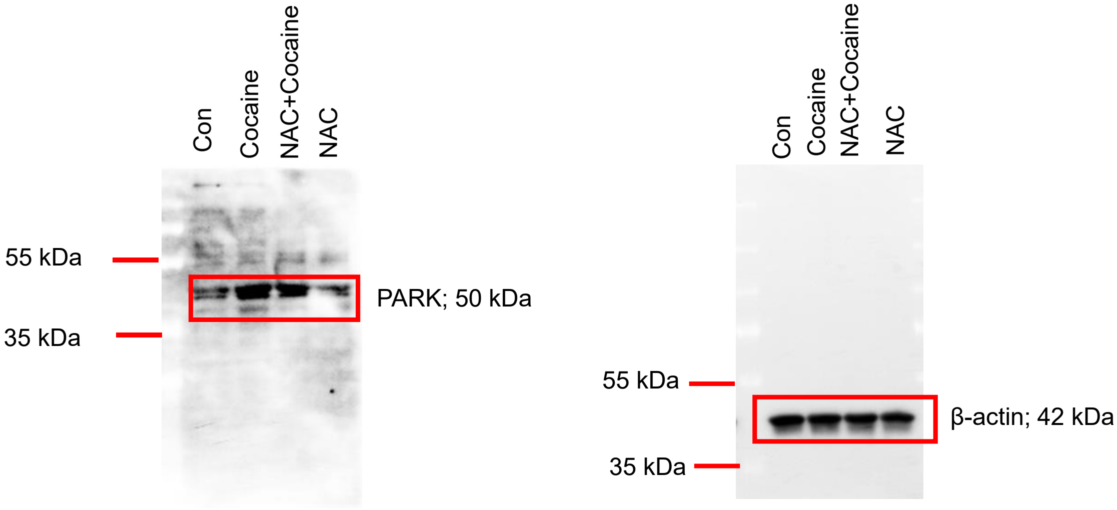

Figure 1D

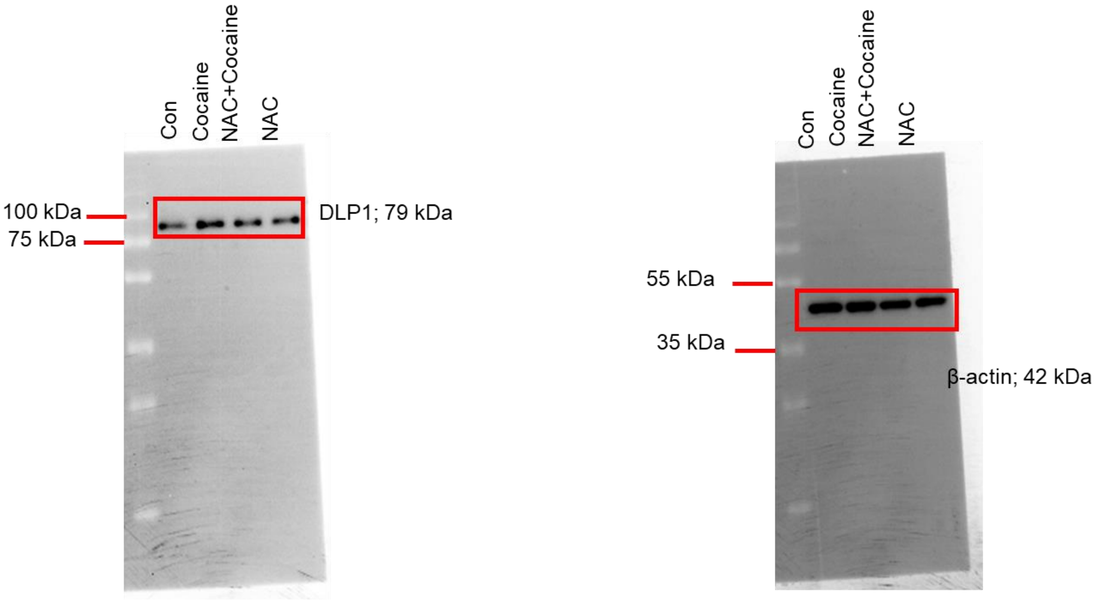

Figure 1E

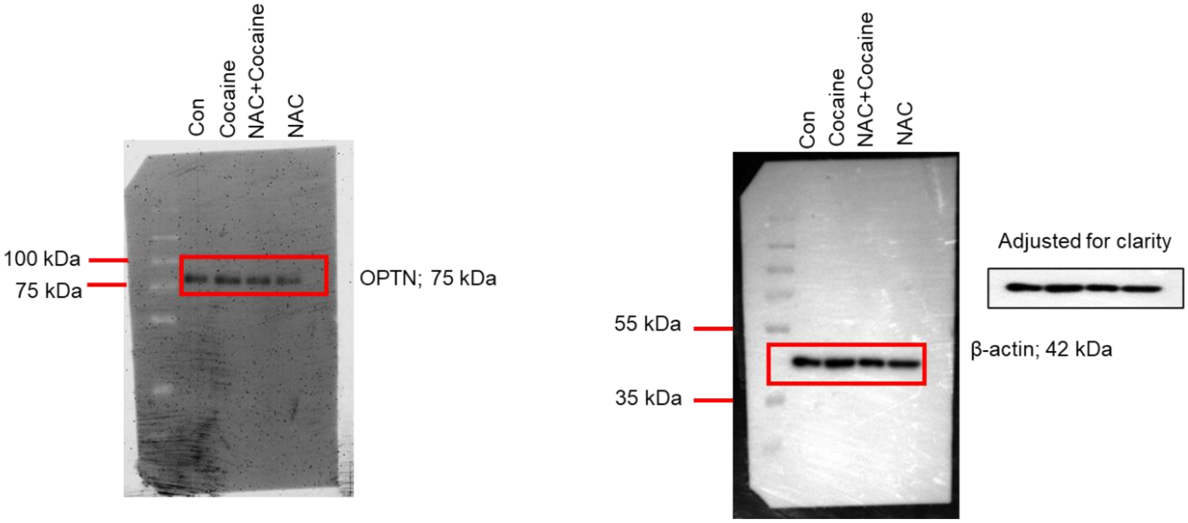

Figure 1F

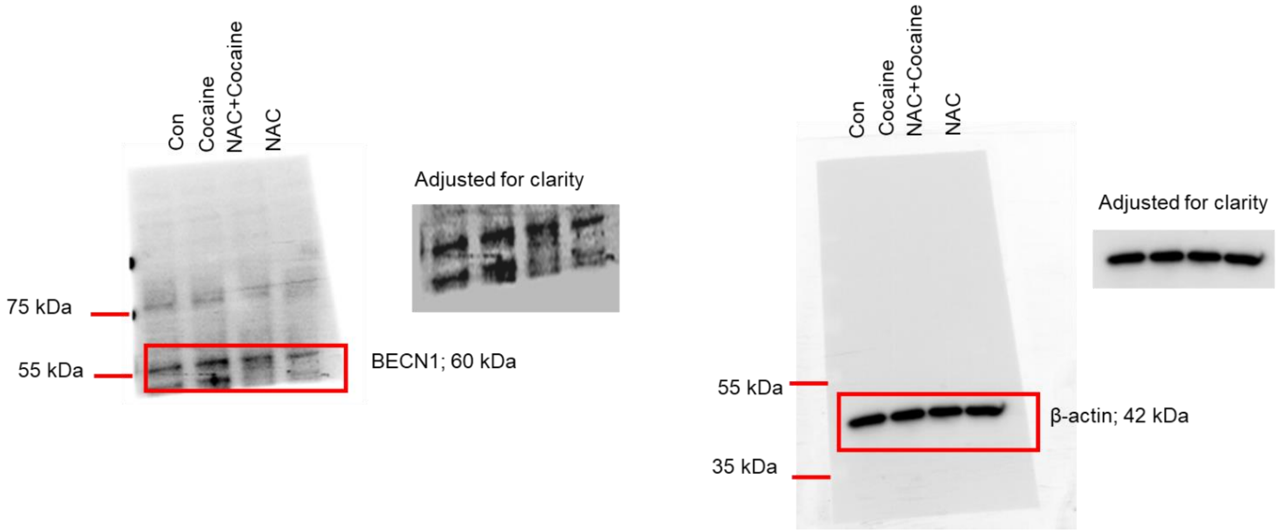

Figure 1G

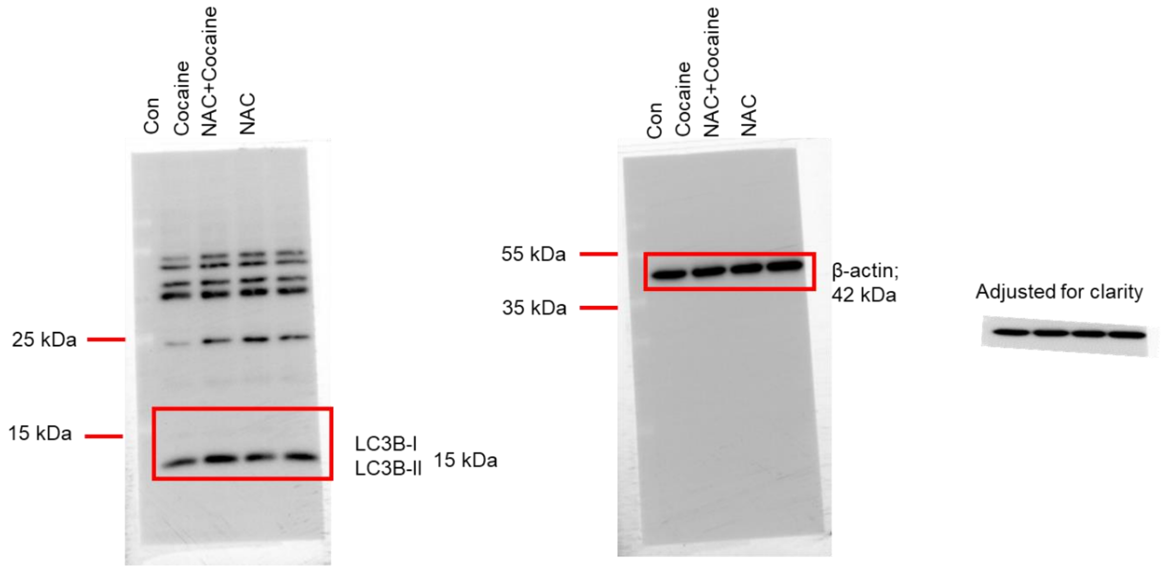

Figure 1H

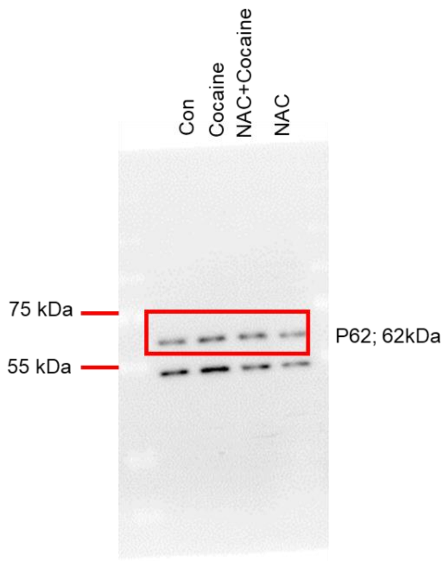
Con
Cocaine
NAC+Cocaine
NAC
75 kDa
55 kDa
P62; 62kDa

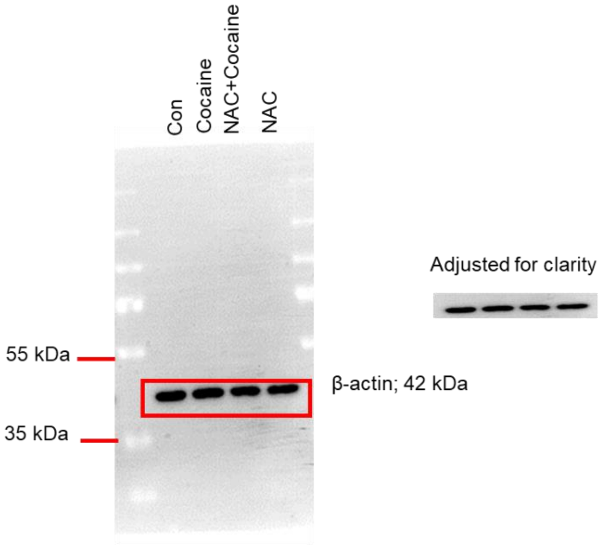
Con
Cocaine
NAC+Cocaine
NAC
55 kDa
35 kDa
β-actin; 42 kDa
Adjusted for clarity

Supplementary Figure S2

Figure 3A

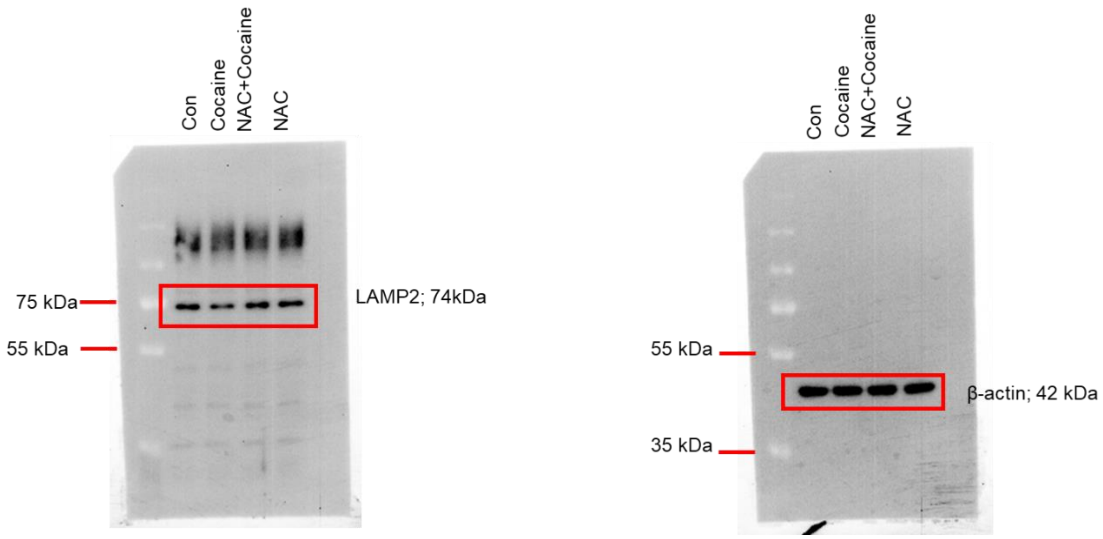

Figure 3B

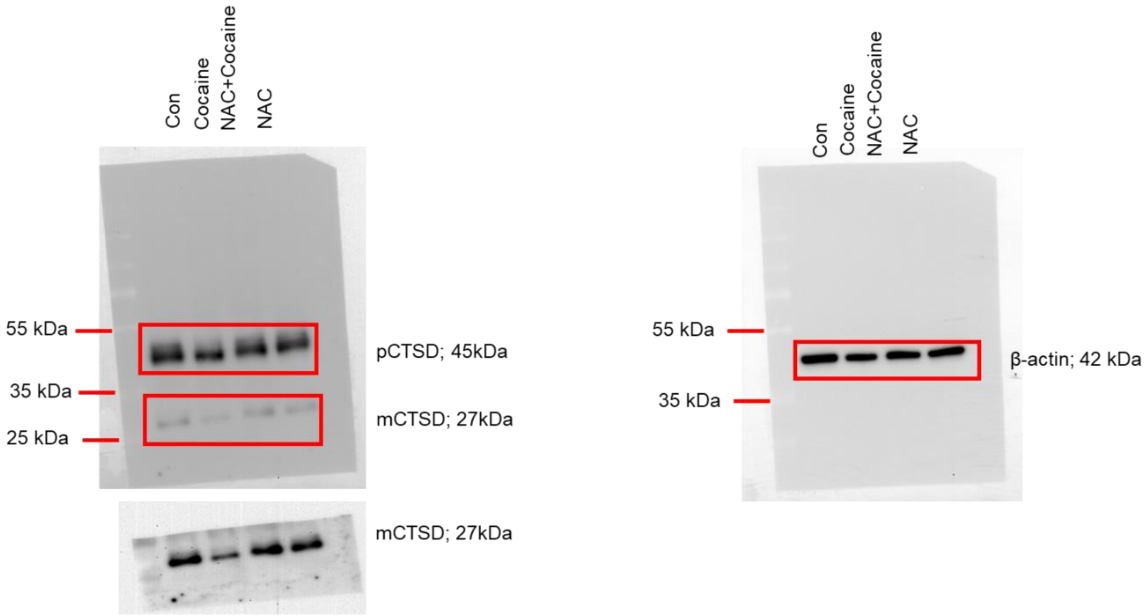

Supplementary Figure S3

Figure 5A

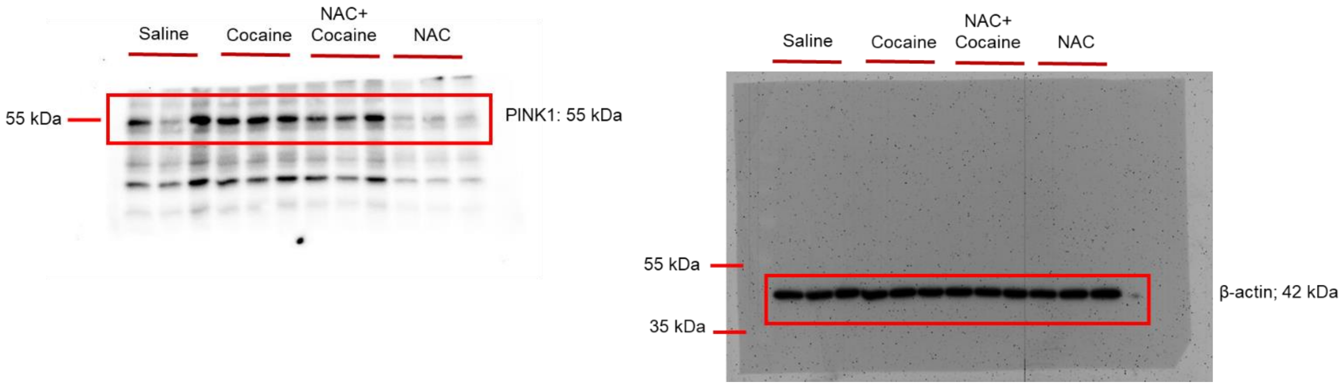

Figure 5B

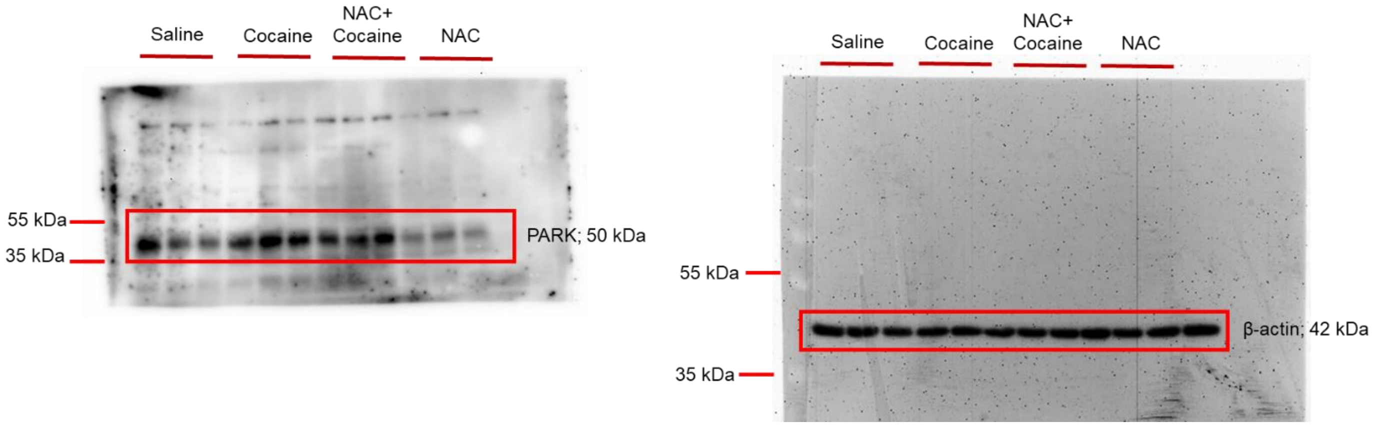

Figure 5C

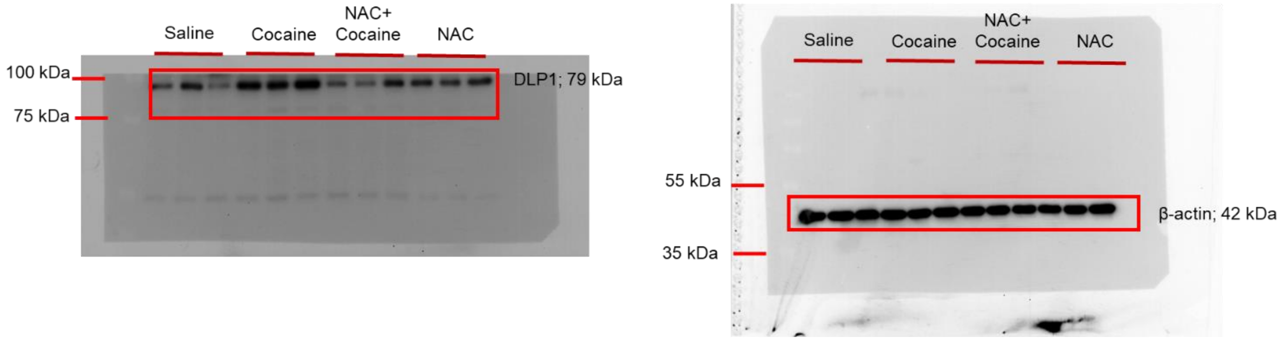

Figure 5D

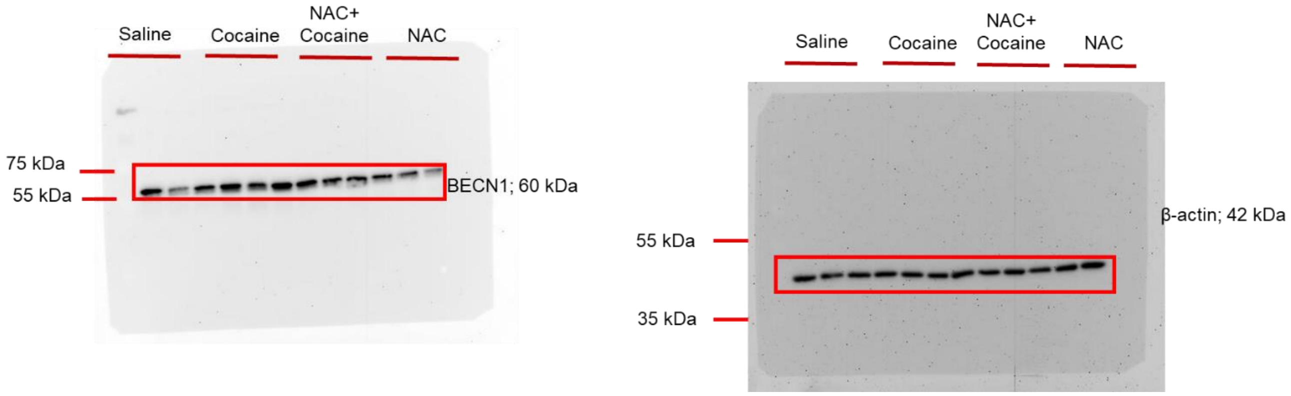

Figure 5E

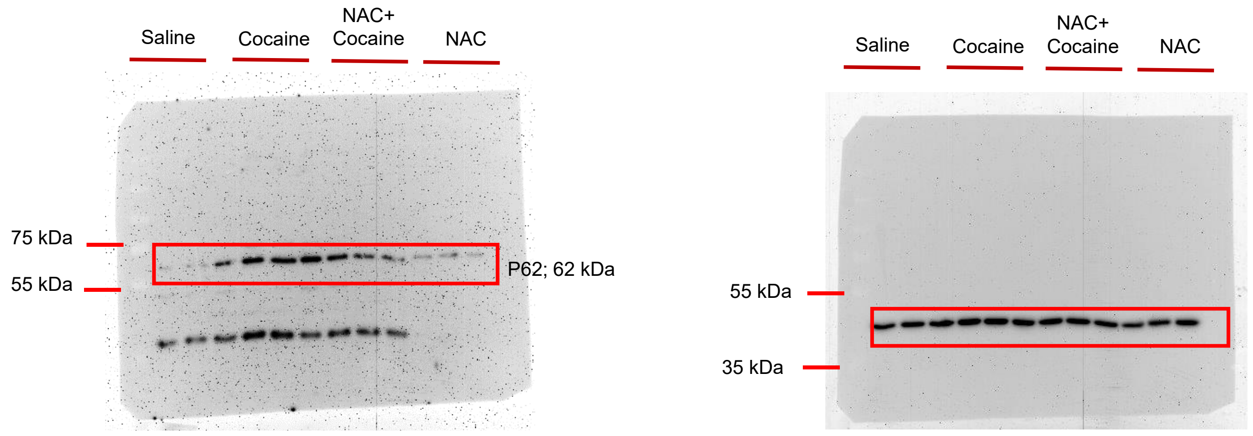

Figure 5F

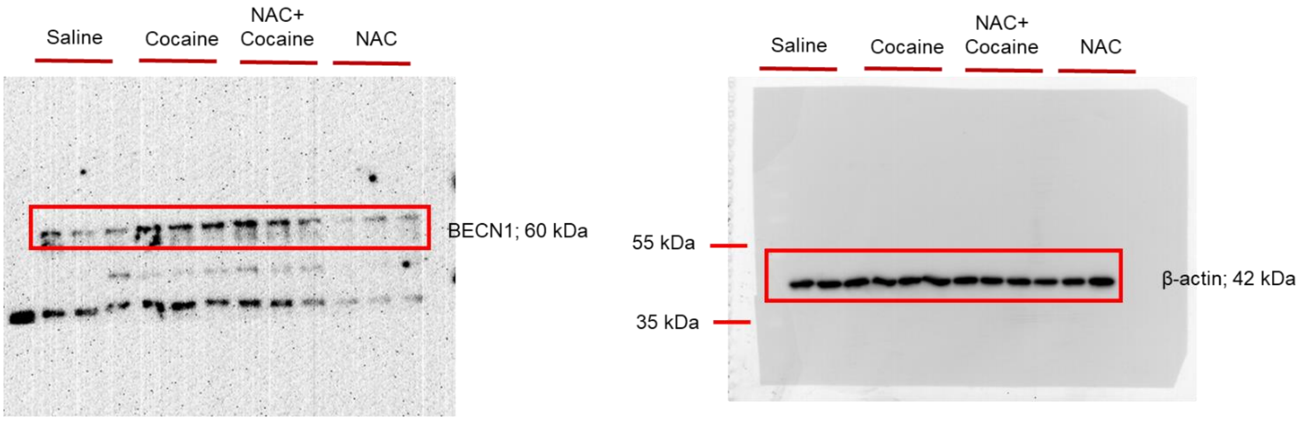

Supplementary Figure S4

Figure 6A

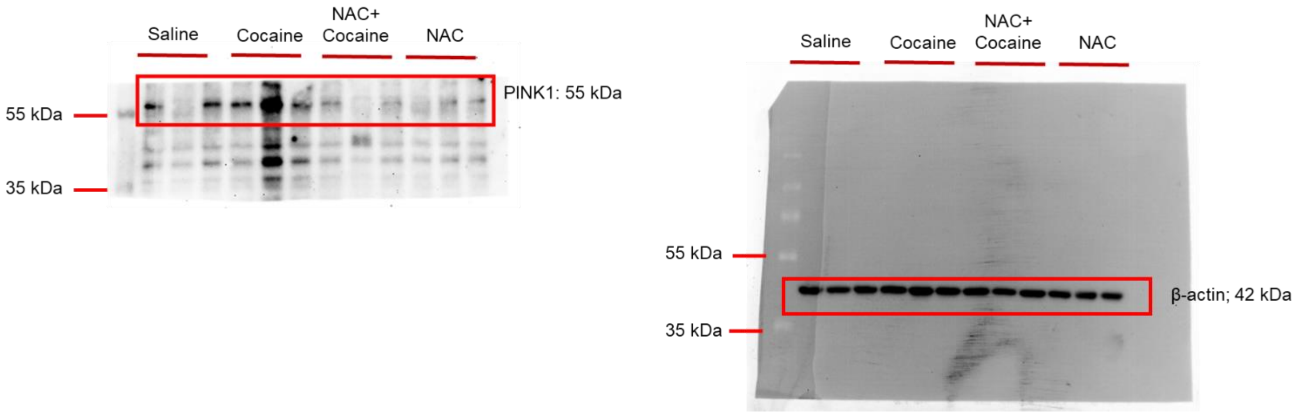

Figure 6B

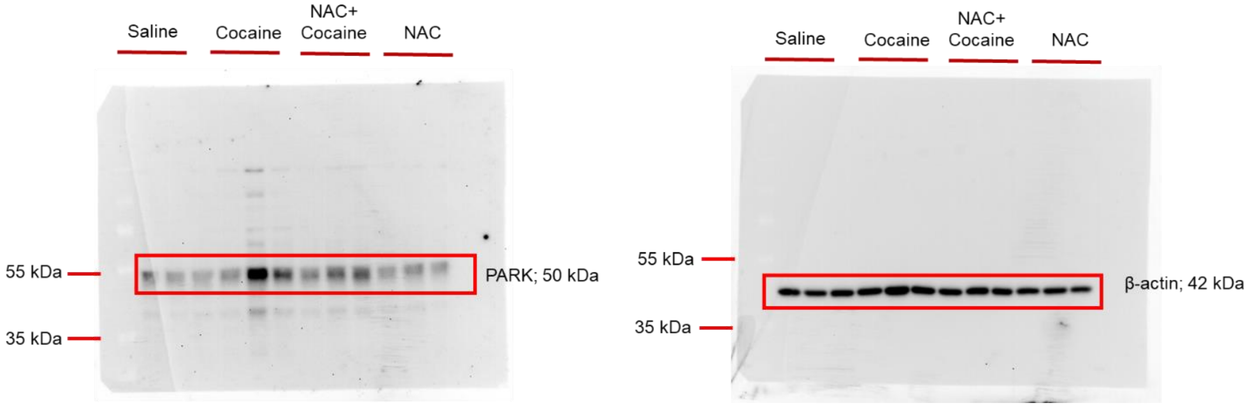

Figure 6C

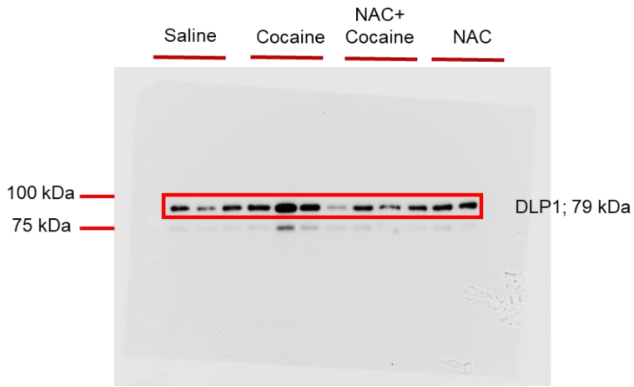

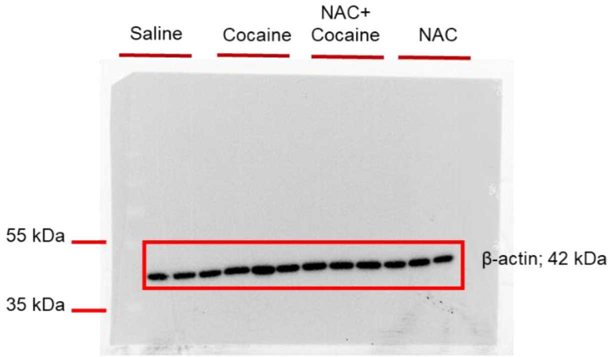

Figure 6D

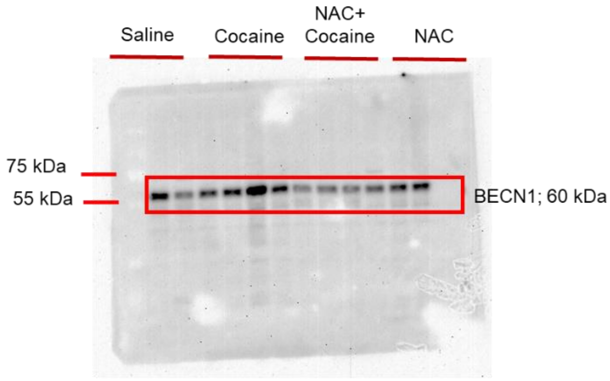

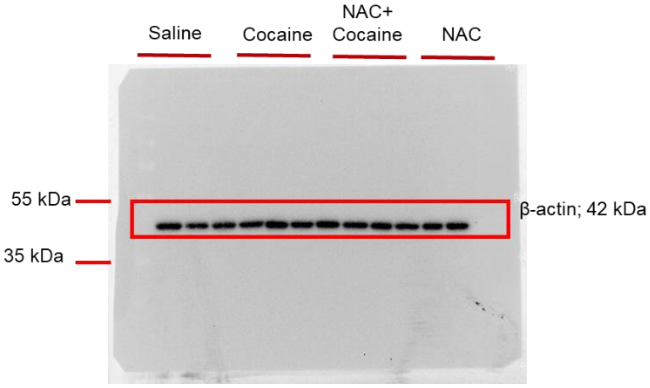

Figure 6E

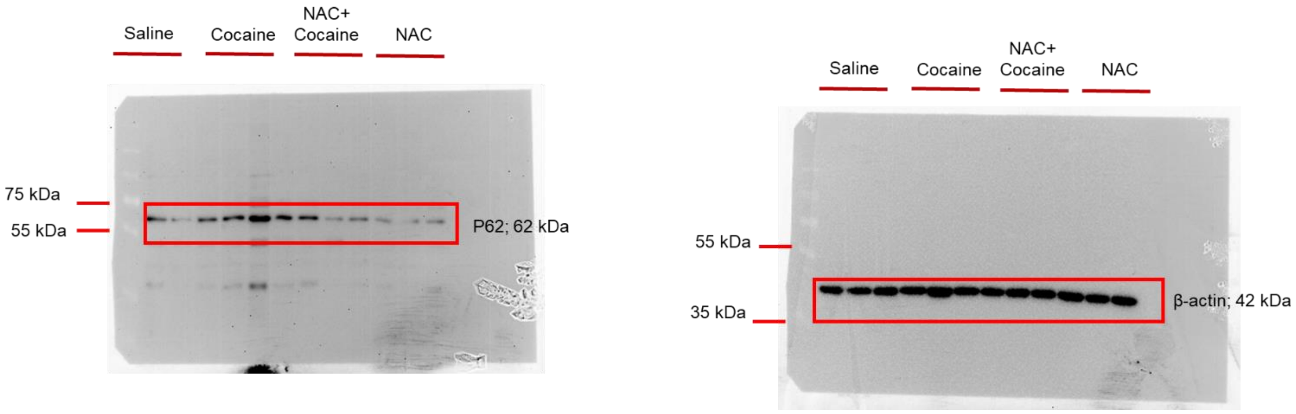

Figure 6F

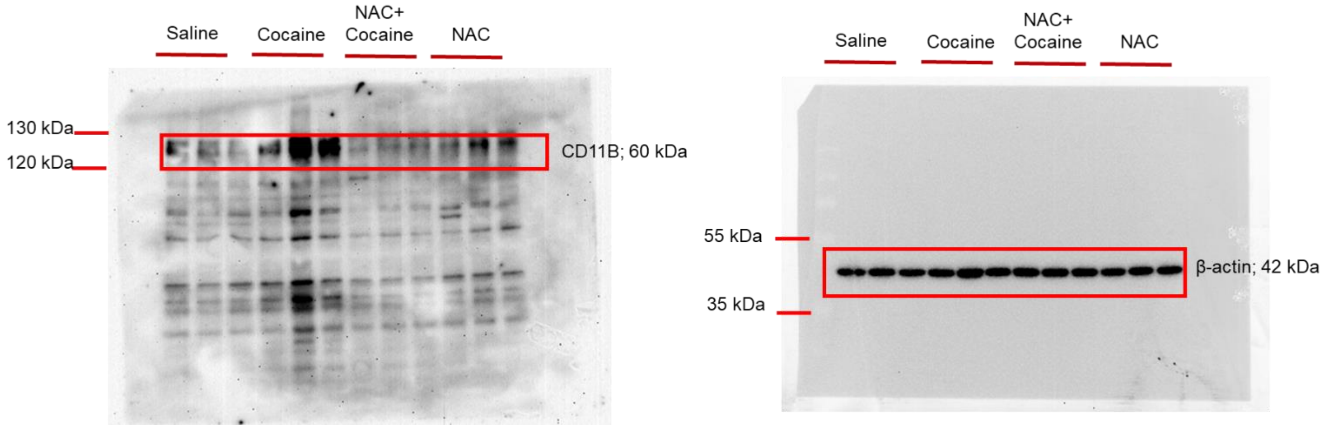

Supplement: Supplementary file 1 [file biology-14-00893-s001.zip › biology-3708341-supplementary.pdf]
